# Evolution of Proliferation and the Angiogenic Switch in Tumors with High Clonal Diversity

**DOI:** 10.1371/journal.pone.0091992

**Published:** 2014-04-14

**Authors:** Scott T. Bickel, Joseph D. Juliano, John D. Nagy

**Affiliations:** 1 Department of Life Sciences, Scottsdale Community College, Scottsdale, Arizona, United States of America; 2 Department of Chemistry and Biochemistry, Arizona State University, Tempe, Arizona, United States of America; 3 School of Mathematical and Statistical Sciences, Arizona State University, Tempe, Arizona, United States of America; Spanish National Research Council (CSIC), Spain

## Abstract

Natural selection among tumor cell clones is thought to produce hallmark properties of malignancy. Efforts to understand evolution of one such hallmark—the angiogenic switch—has suggested that selection for angiogenesis can “run away” and generate a hypertumor, a form of evolutionary suicide by extreme vascular hypo- or hyperplasia. This phenomenon is predicted by models of tumor angiogenesis studied with the techniques of adaptive dynamics. These techniques also predict that selection drives tumor proliferative potential towards an evolutionarily stable strategy (ESS) that is also convergence-stable. However, adaptive dynamics are predicated on two key assumptions: (i) no more than two distinct clones or evolutionary strategies can exist in the tumor at any given time; and (ii) mutations cause small phenotypic changes. Here we show, using a stochastic simulation, that relaxation of these assumptions has no effect on the predictions of adaptive dynamics in this case. In particular, selection drives proliferative potential towards, and angiogenic potential away from, their respective ESSs. However, these simulations also show that tumor behavior is highly contingent on mutational history, particularly for angiogenesis. Individual tumors frequently grow to lethal size before the evolutionary endpoint is approached. In fact, most tumor dynamics are predicted to be in the evolutionarily transient regime throughout their natural history, so that clinically, the ESS is often largely irrelevant. In addition, we show that clonal diversity as measured by the Shannon Information Index correlates with the speed of approach to the evolutionary endpoint. This observation dovetails with results showing that clonal diversity in Barrett's esophagus predicts progression to malignancy.

## Introduction

Natural selection has long been recognized as the ultimate driver of cancer progression and pathogenesis (see [1] for a recent review; see also [2]). In early stages of tumor progression, heterogeneous populations of malignant and healthy cells compete for available resources. Tumor cell clones that have acquired, via mutation and epigenetic effects, malignant “hallmark” phenotypes [3], [4] gain proliferative and (or) survival advantages relative to other lineages in their tumor microenvironment. Eventually the hallmark-carrying mutant clones come to dominate the tumor and destroy tissue homeostasis. If this interpretation is correct, then the mechanism causing malignancy—heritable variation conferring advantages to particular clonal lineages—is precisely evolution by natural selection.

Explaining the adaptive significance of most cancer hallmarks is straightforward. However, angiogenesis—the ability of tumors to generate their own vascular infrastructure—presents a difficult case. Angiogenesis is coordinated directly and indirectly by cancer cells using a variety of signaling molecules, including vascular endothelial growth factor (VEGF), angiopoietins, fibroblastic growth factors (FGFs), platelet-derived growth factor (PDGF), epidermal growth factor (EGF), transforming growth factors (TGF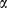 and -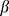), and thrombospondin-1 (TSP-1), among others. These factors act in a variety of ways on vascular endothelial cells and (or) their precursors. Target cell responses include proliferation, chemotaxis and differentiation into functional microvessel endothelial cells [3]–[6]. The balance between pro- and anti-angiogenic molecules in the local milieu define the “angiogenic signal” [7]. In hypoxic tissues, this balance tips in favor of angiogenesis [5], [6]. Cancer is often characterized by derangement of this signaling system, generating among certain tumor clones more-or-less constitutive production of pro-angiogenic signals and receptors, a condition referred to as the angiogenic switch [3], [4], [7]–[12]. The intensity of this switch varies among tumors even of the same histological type and tissue of origin [4], [13].

Angiogenesis clearly benefits tumors. In addition to nutrient delivery and waste removal, tumor microvessels provide routes for metastasis. However, all tumor cells receive the benefits of angiogenesis whether or not they participate in producing the signal. Therefore, the signal is a public good. As is well known from decades of research into the “free-rider” problem in economics and evolutionary biology, public goods are susceptible to exploitation by free-riders. In this context, free-riders would be clones that, by mutation or epigenetic alteration, decrease or stop their own production of proangiogenic signals. Since metabolic energy is required to produce the angiogenic signal, free-riders eliminate one drain on internal energy reserves with no immediate detriment. However, they gain an immediate advantage—saved energy reserves can be committed to proliferation and maintenance metabolism. Free-rider clones would therefore be expected to expand more rapidly than angiogenic clones due to their inherited advantage. The obvious fact that the tumor, and the free-riders themselves, would suffer hypoxia once free-riding becomes dominant is irrelevant. Natural selection does not act to benefit the tumor. Selection simply favors clones with the highest growth and survival potential once the chains of kin selection and other evolutionary forces compelling cooperation have been broken. In any environment, even severely hypoxic ones, free-rider clones will always have an advantage over angiogenic clones, all else being equal, because they have less demand for energy to produce a public good. Any angiogenic clone will certainly benefit from being angiogenic. But the free-rider benefits equally. The fact that cancer cells tend to disperse from unfavorable environments does not eliminate the problem. It simply spreads it. If the hallmarks of cancer are consequences of evolution, it is not immediately clear why the angiogenic switch persists in malignant tumors.

Indeed, modeling studies initiated by one of us (JDN) predict that such nonangiogenic free-riders can damage or perhaps destroy all or part of a growing tumor [14]–[16]. This predicted “tumor-on-a-tumor” phenomenon has a conceptual sister, *viz*. hyperparisitism—one parasite exploiting another. Therefore, the parallel term “hypertumor” was suggested to describe it [14], and it has since been recognized as a form of evolutionary suicide [17], [18].

**Figure 1:**
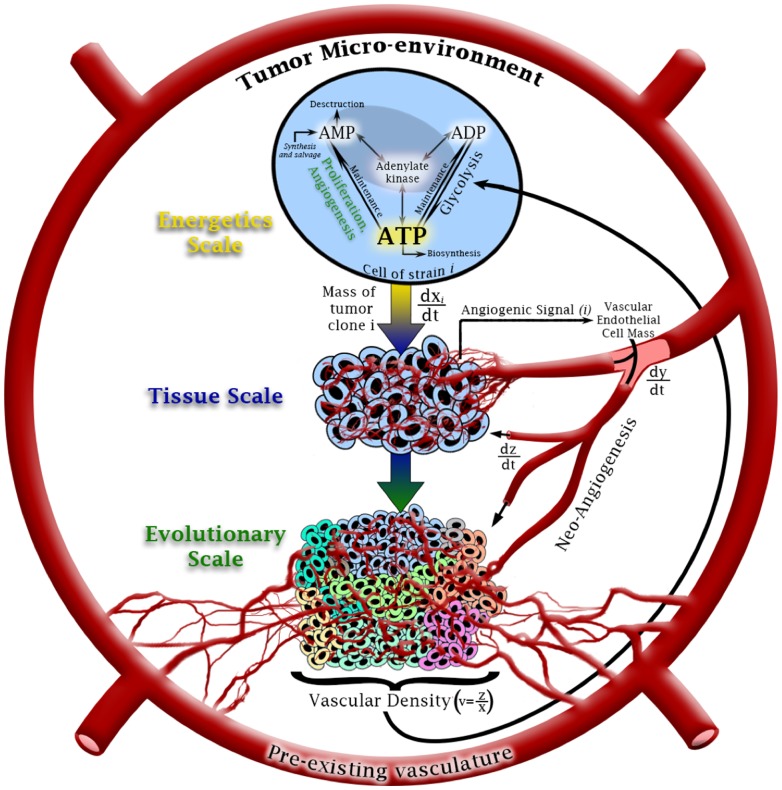
Schematic representation of the multiscale model. The “energetic scale” submodel [equations (1) through (10)] governs dynamics of adenylate (AMP, ADP and ATP). Interconversions among the three species occur via maintenance metabolism (e.g., biosynthesis, volume control), chemical energy to support proliferation and angiogenesis signal production, glycolysis and, most importantly, the adenylate kinase reaction, among others. Sources for adenylate include *de novo* synthesis of AMP and salvage from nucleic acid catabolism. Adenylate sinks include AMP destruction by deaminases and nucleotidases and ATP loss to nucleic acid synthesis. Clonal expansion or regression at the tissue scale [model (11)] depends primarily on mean tumor microvessel density, which is controlled in part by angiogenic factor secretion by existing clones. Blood vessels grow from existing vasculature via chemotaxis and maturation of vascular endothelial cell precursors in and near the tumor. At the evolutionary scale, angiogenic and proliferative potential varies among clones (different colored cell subpopulations) as they compete for resources delivered by microvessels. Evolutionary scale dynamics are handled in the simulation (see “Simulation Methods” above).

The early hypertumor models were limited by the fact that costs associated with hallmark phenotypes could not be investigated because the models lacked a proper description of energetic trade-offs. That limitation was addressed in a recent study by Nagy and Armbruster [17] in which the original models were extended to include an energetic “opportunity cost.” This extension required the addition of a submodel describing intracellular adenylate dynamics to the existing tissue-level model of angiogenesis. The result was a multiscale system with three distinct spatial and temporal levels: intracellular energy metabolism on scales of µm and seconds to minutes; tissue-level on scales of mm and hours to days; and evolutionary, with scales of cm and months to years (Fig. 1). In this formulation, ATP, the primary energy currency in cells, is partitioned among three major energetically demanding programs: proliferation, cell maintenance and secretion.

This model confirms that evolution of hallmarks acting on proliferation rate differs markedly from evolution of the angiogenic switch. In particular, selection drives energy allocation for proliferation to an intermediate state that balances evolutionary benefits of reproduction with opportunity costs of shunting reducing power away from cell maintenance. This attracting state is an evolutionarily stable strategy (ESS) *sensu* Maynard Smith and Price [19], [20]; that is, it is a strategy that, when used by almost all residents of a population, cannot be invaded by any possible mutant. This ESS is also convergence stable (essentially an evolutionary attractor; see [21] for a review).

In contrast, angiogenic potential does not evolve to an intermediate state in this model. As predicted by the free-rider argument, direct selection on angiogenesis signaling is powerless to produce the angiogenic switch via the benefits of eventual increased perfusion. However, this model predicts an indirect evolutionary pathway to the angiogenic switch caused by an interesting property of the adenylate homeostasis mechanism. Both modeling and *in vivo* studies suggest that intracellular equilibrium ATP concentration is a unimodal function of overall cell metabolic rate [11], [23]. To the left of the mode (relatively low metabolic rates), cells respond to slight increases in ATP consumption by excessively ramping up *de novo* adenylate synthesis, resulting in a paradoxical increase in equilibrium ATP concentration. Therefore, under conditions favoring such overcompensation, mutants that increase production of the angiogenic signal, which requires ATP for protein synthesis and secretion, can gain more ATP for proliferation. This pleiotropic effect on both proliferation and angiogenic potential confers to the mutant a selective advantage. In the model, the evolutionary picture is complicated by an interaction between this phenomenon and the neovascularization in response to the angiogenic signal. This interaction generates an ESS that is always evolutionarily repelling (details in [17]). That is, if clones differ only in ATP allocation to angiogenesis, clones slightly more (or less) committed to angiogenesis than the ESS are vulnerable to invasion by a mutant clone with higher (respectively, lower) energy allocation to angiogenesis. Therefore, this model predicts that, given enough time, selection will run away either to vascular hyper- or hypoplasia, eventually reaching a tumor inviability region. The latter possibility is the original hypertumor prediction, while the former represents a novel form of evolutionary suicide [14], [16], [17].

The open questions we address here are the following: (i) what are the likely trajectories tumors traverse through their evolutionary “strategy spaces” as angiogenic and proliferative potentials evolve? (ii) what variation in these evolutionary trajectories can be expected? and (iii) how rapidly will the traverse occur? Answers to these questions are required before practically testable predictions from the model can be distilled, but they could not be addressed in the previous modeling attempts. In these studies, the evolutionary analysis relied on the techniques of adaptive dynamics [21], [24], [25], which require the assumption that mutation dynamics are much slower than ecological dynamics. At most only two competing clones can exist in a tumor. One arises as a rare mutant within a tumor populated almost exclusively by a resident clone. Competitive exclusion is the rule in these pairwise bouts; either the mutant invades the tumor and eliminates the resident clone, or the resident eliminates the mutant. Either way, “ecological” dynamics of competition are assumed to reach their endpoint before a new mutant arises, so ecological and evolutionary timescales decouple. Also, all mutations are assumed to have a small effect; therefore, the difference between phenotypes of resident and mutant clones is always small [25]. Given the genomic instability characteristic of many malignant tumors [4], these assumptions are likely to be violated in real tumors. So, here we repeat the evolutionary analysis with the adaptive dynamics assumptions relaxed. To achieve this, we first define a stochastic simulation analogue of the multiscale evolutionary model in [17]. The equations governing intracellular adenylate, tumor growth and angiogenesis are unchanged. The only alteration we introduce is at the evolutionary scale. In particular, we allow an indefinite number of clones to compete at the same time, and mutant clones arise at random times independent of the current state of the system. Here we show that relaxation of the adaptive dynamics assumptions has no effect on predicted evolutionary endpoints from the original adaptive dynamics analysis. However, the simulations predict that evolutionary dynamics of both angiogenesis and proliferative capacity is dominated by mutational history. Practically, this prediction suggests that the disease is on an evolutionary transient throughout its clinical course—that is, an attracting ESS is rarely if ever approached—and the tumor's evolutionary tempo and trajectory are largely determined by phenotypes of early mutants, which in practice are likely to resist prediction. We refer to this prediction as the historical contingency effect, following [26].

## Methods

The deterministic model underlying our simulations [17] comprise two distinct systems of ordinary differential equations (ODEs) governing dynamics at three time and spatial scales (Fig. 1). The first system, which extends the pioneering work of Martinov, Ataullakhanov, Vitvitsky and colleagues [22], [23], tracks intracellular adenylate dynamics, with adenylate concentrations scaled in fmol and time scaled in mins (“Energetics Scale” in Fig. 1). The second system, operating at tissue and evolutionary scales, describes growth dynamics of the tumor, dynamics of its vascular infrastructure and competition among clones within the tumor. The tumor is assumed to comprise some number of genetically and phenotypically distinct clones, a collection of immature vascular endothelial cell precursors (VECPs) and patent, functional microvessels. In the original formulation, the number of competing clones was limited to 2, but here we allow the tumor to house an arbitrary number, 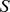, of distinct clones. Dynamics of a single clone (“Tissue Scale” in Fig. 1) is governed by two ODEs—one for clonal mass (in g) and one for mean tumor microvessel density. Time is scaled in days. Overall tumor dynamics (“Evolutionary Scale” in Fig. 1) is therefore determined by 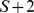 ODEs, one for each clone, plus equations for VECPs and microvessels, with dependencies on the energetic states of cells determined by equations at the energetics scale. This tissue-level model is derived directly from previous work of Nagy and colleagues [14], [16].

Interactions among all three scales revolve around tumor perfusion, measured as microvessel length density. In the model, hypoxic tumor cells secrete a chemical signal composed of a variety of tumor angiogenesis factors (TAF) to which VECPs respond by proliferating, maturing and integrating themselves into functional microvessels. The interaction between intracellular and tissue levels arises as cells partition their available chemical potential energy, primarily in the form of ATP, among three energy-dependent activities: maintenance metabolism (cell volume regulation, maintenance protein production and other life-support physiology), proliferation and, potentially, secretion of TAF. In turn, these three activities feed into tissue-level phenomena of blood vessel growth and clone-specific expansion. These growth phenomena then feed back to the intracellular scale because relative growth rates of vessels and tumors determine perfusion and therefore nutrient delivery, which in turn sets the cellular energy charge and ATP regeneration rate, as detailed below. Tumor vascular dynamics depend on both the strength of the angiogenic signal and rate of tumor growth. Tumor vascular density determines rate of ATP synthesis. Since malignant tumors are often characterized by dampened oxidative metabolism (Warburg effect [27], [28]), vascular feedback on ATP synthesis primarily occurs via delivery of glucose for glycolysis (Fig. 1). Although an exhaustive derivation of the model can be found in [17] (see also [14]), we provide a detailed outline below for completeness. All parameters in the model have been estimated carefully from data when possible or from biological first principles and model behavior when not. Details of the prarameterizations are outside the scope of this paper but are available in references [14], [16], [17].

### Cell Energetics Scale Model

Let 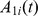, 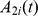 and 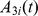 represent mean intracellular concentrations of adenylate 5′ mono-, di- and triphosphate, respectively, in clonal lineage 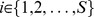 at time 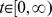. (Variables and parameters in this model are summarized in Tables 1 and 2, respectively.) We assume that each clone acts independently of all others, and all cells in a given lineage are identical. Adenylate concentrations are to be understood as mean-field or ensemble averages within clone 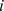.

**Table 1:** Dependent variables studied in this model.

Variable	Meaning
*t*	Time
*A_j_*(*t*)	Concentration of adenylate 5′ *j*-phosphate
*x_i_*(*t*)	Mass of the *i* ^th^ clone's parenchymal cells
**x**(*t*)	Total parenchymal mass
*y*(*t*)	Total mass of precursor VECs
*z*(*t*)	Total length of mature microvessels
*v*(*t*)	Tumor vascularization ( = *z*/**x**)

**Table 2:** Parameters and default values representing a resting cell (from [17]).

Parameter	Meaning	Default	Units
**Evolutionary**			
*λ*	Mutation rate parameter	0.1	hr^−1^
*η_i_*	Proliferation secretion effort of clone *i*	1, max = 12	min^−1^
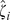	Basic TAF secretion effort of clone *i*	0.11	g/U/min
**Energetic**			
*α_a_*	*de novo* AMP synthesis rate	5.725×10^−5^	fmol/min
*k*	Adenylate kinase rate parameter	10^6^	1/fmol/min
*β*_1_	Maintenance ATP to AMP rate	4	min^−1^
*β*_2_	Maintenance ATP to ADP rate	4	min^−1^
*γ_a_*	Adenosine kinase rate	0.01	min^−1^
*μ*	ATP destruction rate	0.01	min^−1^
*ξ*	Nutrient sensitivity of TAF secretion	10/3	g/U
*s_max_*	Physiological max ATP regeneration rate	390	min^−1^
*M*_1_	AMP deaminase parameter	0.4	fmol/min
*M*_2_	Nucleotidase parameter	9.167×10^−7^	fmol/min
*k*_1_	AMP deaminase parameter	0.5	fmol
*k*_2_	Nucleotidase parameter	5×10^−3^	fmol
*k*_3_	Nucleotidase parameter	2.5×10^−10^	fmol^2^
*k*_4_	Nucleotidase parameter	5×10^−5^	fmol
**Tissue**			
*p*	Basic parenchyma proliferation rate	0.072	hr^−1^
*k_s_*	Proliferation sensitivity parameter	2	fmol/min
*m*	Parenchyma mortality parameter	0.0698	fmol/hr
*α*	Max VEC response to TAF	0.1	hr^−1^
*k_v_*	Sensitivity of VECs to TAF	0.0115	fmol/min
*β*	VEC death/maturation rate	0.04	hr^−1^
*γ*	VEC maturation rate	3	U/g/hr
*δ*	Microvessel remoldeling rate	4×10^−3^	g/U/hr

Intracellular adenylate dynamics are governed by the following system of ODEs:
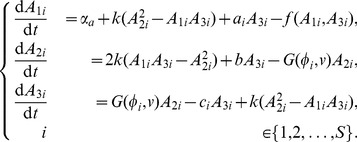
(1)Total adenylate in cells is controlled primarily via synthesis and destruction of AMP. In the model, AMP appears *de novo* at constant rate 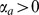, representing mainly synthesis from inosine monophosphate and salvage from nucleic acid recycling. The function 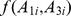 represents irreversible AMP recycling by enzymes primarily in the 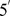 nucleotidase and AMP deaminase families. Specifically,
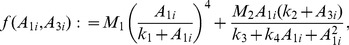
(2)which is an empirical model of AMP destruction suggested by Martinov et al. [23], who also provided empirical estimates of (positive) parameters 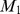, 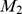, 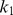, 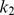 and 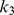. The first and second terms in equation (2) represent the actions of AMP deaminases and 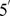 nucleotideases, respectively.

In resting mammalian cells, adenylate dynamics are dominated by the adenylate kinase reaction,
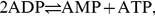
(3)which is represented in the model by the second, first and third terms in each equation of model (1), respectively. In most cells this reaction has approximately equal forward and backward rates [29], which we denote here as the positive constant 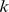.

Besides the adenylate kinase reaction (3), interconversion among adenylate species revolves around either ATP hydrolysis or synthesis. The former is primarily governed by positive parameters 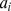, 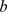 and 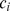, which themselves are sums of constants representing metabolism supporting proliferation, TAF secretion and maintenance. In particular,
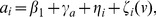
(4)

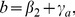
(5)

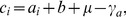
(6)for 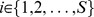. Parameters 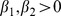 are basic maintenance metabolism rates. The former is the ATP→AMP conversion rate, e.g., for biosynthesis (amino acid adenylation) and to power the phosphoribosyl pyrophosphate synthetase reaction, among others [17]. The latter (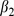) represents the ATP→ADP conversion rate, primarily, but not exclusively, for cell volume control. A second pathway from ATP to both ADP and AMP exists via the adenosine kinase reaction,
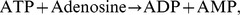
which we assume occurs at base rate 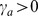. Parameter 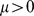 is the per-ATP rate of nucleic acid synthesis (assumed to be an irreversible sink for ATP). All these parameters are assumed constant across clones.

The key evolutionary parameters are 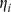 and 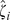, both positive constants representing mean per-molecule rates at which clone 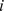 cells allocate ATP to proliferation and angiogenesis secretion, respectively. The dependence of 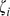 on 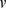 arises because clones vary the intensity of their angiogenic signal as a function of vascular density. Specifically, we assume that
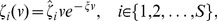
(7)where 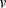 is vascular density (defined in the Tissue Scale section below). This functional form, adapted from [14], assumes that TAF signaling rate is a unimodal function of vascular density, 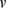. It qualitatively mirrors observed increases in secretion of angiogenesis-promoting growth factors as cells become hypoxic [5] with the added assumption that cells suffering extreme hypoxia lose the ability to produce the signal. Parameter 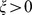 is constant across clones and determines the vascular density at which angiogenesis secretion peaks. Parameter 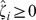 varies among clones, is constant within a clone, and measures the general intensity of the angiogenic signal.

Finally, ATP is “regenerated” from ADP primarily via glycolysis in cancer cells. This conversion is governed in the model by the function 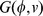. Here, 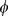 denotes the “energy charge” of a cell, which is a sort of weighted average of “high energy” phosphoryl groups in adenylate. Specifically,
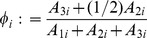
(8)is the mean energy charge of cells in the 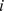
th clone. Mean glycolysis rate is assumed to vary among clones only as they vary in mean energy charge; in particular,
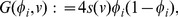
(9)where
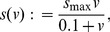
(10)and 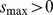 is constant. The particular forms and parameterizations for 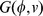 [equation (9)] and 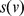 [equation (10)] were chosen to fit data from [22] (see [17] for details).

### Tumor Tissue Scale Model

Let 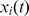, 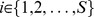, be the mass (in g) of clone 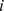 at time 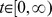. Also let 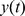 and 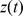 be VECP mass (in g) and total length of microvessels, respectively, within the tumor at time 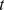. Microvessel length is scaled such that 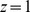 when total length of microvessels in the tumor equals that of 1 g of healthy tissue in the tumor's site of origin [14], [17]. We assume that mean proliferation and angiogenesis signal production for cells of clone 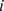 depend on the clone's mean intracellular ATP concentration. However, mean ATP concentration depends on vascular density, 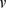, the clone's energy commitment to proliferation, 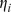 and its commitment to angiogenesis, 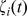 (see previous section). Therefore, there is a continuous feedback between energetic and tissue scale dynamics. Since adenylate dynamics of model (1) equilibrate very rapidly on the time scale of the tissue model (11) (ref. [17]), we further assume that mean ATP concentration in all clones is locked in quasi-equilibrium for the adenylate model. We denote the ATP quasi-equilibrium for the 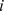
th clone as 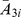.

These assumptions lead to our tissue-level model of tumor growth, adapted from [14], [17]:
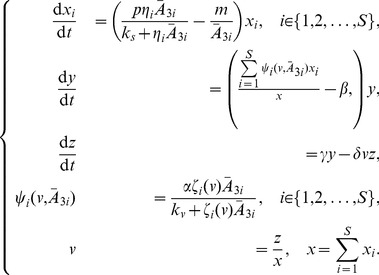
(11)(Note that we have suppressed the time arguments of dependent variables for clarity.) Here, 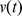 denotes tumor microvessel length density (in microvessel units/g), and 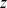 is scaled such that 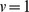 when tumor vascular density equals that of surrounding healthy tissue. The mean ATP hydrolysis rate in support of proliferation in clone 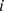 is 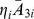. Mean per-cell proliferation rate is a monotonically increasing, saturating function of 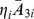, which we represent with a Michaelis-Menten form in which 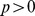 and 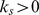 are maximum proliferation rates and half-saturation constants, respectively. We also assume that a clone's mean per capita mortality rate is inversely proportional to 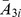, with constant 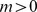.

Mean energetic commitment to angiogenic signal production in clone 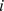 takes a similar form to that for proliferation, *viz.* 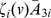. As with proliferation, ATP invested in angiogenesis gives diminishing returns, as represented by another Michaelis-Menten function, with maximum angiogenic signal production 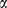 and half-saturation constant 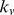 [see equation for 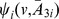 in system (11)]. Overall angiogenic signal is the average signal strength of all clones weighted by clone density, and we assume that per capita proliferation of VECPs is proportional to the strength of this signal [first term, second equation in system (11)]. Mean per capita VECP mortality and maturation rates, combined into parameter 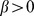, are assumed to be fixed. As VECP cells mature, they integrate themselves into functional tumor microvessels at rate 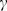, composed of both the rate constant and a unit conversion factor. There is evidence that tumors actively maintain their vascular infrastructure even after its initial construction [5], [6], [12], [30]. Viewing this maintenance as the tumor provisioning microvessels with a resource, which may simply be space, we assume that this resource is proportional to tumor mass, say 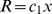, where 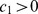 is constant. Resource availability per microvessel unit is therefore the ratio 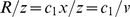. We assume that per capita microvessel remodeling rate is inversely proportional to this ratio; that is, it is proportional to 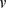 with proportionality constant 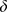 (which includes 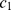; second term, 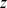 equation of system (11); see also ref. [14]).

This modeling approach implicitly assumes that the average conditions in the tumor are predictive of tumor dynamics. In particular, vascularization, 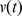, is interpreted as mean vessel density throughout the tumor at any given time (or, alternatively, the ensemble average of many similar tumors). However, since mutant clones are initially localized and vary in angiogenic efficacy, one should question the assumption that all clones are equally vascularized on average. On the other hand, cancer cells are characterized by their ability to infiltrate surrounding tissues, and many if not most exhibit positive chemotaxis up nutrient gradients and therefore tend to move towards areas of locally high vascularization [31]. So how violated the averaging assumption is, and the consequences of that level of violation, remain open questions to be addressed in subsequent approximations.

### Evolutionary Scale Model

The tumor's vascular support, measured by 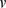, reacts to changes in the clonal composition of angiogenic phenotypes, their prevalence and overall abundance. In turn, clonal composition is determined by selection pressures generated by a particular vascular environment. This interaction dictates dynamics at the evolutionary scale. Here we follow the prevalence of each phenotype within the tumor, where the phenotype of clone 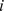 is defined as 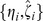; that is, the phenotype is the clone's energetic commitment to angiogenesis and proliferation. We make no explicit hypotheses about how these phenotypes relate to genotypes except for the general assumptions that these traits have high heritability, that they are polygenic, and that new phenotypes may arise by mutation. However, we allow the possibility that multiple genotypes can generate the same phenotype. We also leave open the possibility that phenotype could result from a persistent epigenetic change. For simplicity, however, we refer to new phenotypes as “mutants.” In any case, phenotype is fixed for all cells in the clone, although angiogenesis signaling and proliferation rates in a single clone are not fixed since these depend on vascularization, which is dynamic.

Evolutionary dynamics of clonal phenotypes in this model were initially analyzed using adaptive dynamics [17]. The technique is founded on the question, can a rare mutant strategy invade an otherwise monomorphic population using a different strategy (the “resident” strategy) in the resident's equilibrium environment [21], [24], [25]? The set of all possible strategies is referred to as the strategy space. One analyzes the ability of any possible mutant phenotype to invade any resident strategy in an environment set by the resident. Such an analysis produces a good, if not complete, picture of the evolutionary dynamics, including the existence and location in strategy space of evolutionarily important points or sets. In particular, adaptive dynamics identifies evolutionarily stable strategies (ESS), can be used to determine whether these points are evolutionarily attracting (continuously stable strategies [32]) or repelling and assess potential for evolutionary suicide [18] and evolutionary branching [21], among other things.

However, adaptive dynamics is limited by two fundamental assumptions. First, interarrival times between mutations must be long compared to population dynamics so that fate of the mutant is determined before the next mutant arises. Although this assumption improves analytical tractability, it removes mutational dynamics from the evolutionary picture, rendering transient evolutionary dynamics invisible. We can only see the potential evolutionary endpoints. Also, this assumption is almost certainly violated in most cancers, which are well-known to be genomically and genetically heterogeneous [1], [33]. Second, most adaptive dynamics analyses rely on the assumption that mutations have small phenotypic effects. Although this assumption is not strictly required, relaxing it typically compounds analytical complexity. But again, in cancer this assumption has dubious validity because even minor mutations in both coding and control regions of genes can have massive effects on cell phenotype. A relevant example here would include a mutation in the control region of *HIF1A*, the gene for the 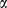 subunit of hypoxia-inducible factor 1, which could generate an enormous alteration of a clone's angiogenic potential [34]. The main goal of this paper is to assess the effects these assumptions have on the predictions of the coupled models (1) and (11). Therefore, we relax these assumptions, at the cost of sacrificing analytical tractability, which leads to the simulations described below.

### Simulation Methods

In concept, our simulations operate as follows. Initial tumors are assumed to be small (10 mg) and monomorphic with vascular density equal to that of surrounding healthy tissue (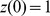). Therefore, simulation initial conditions were always the following: 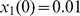 g, 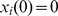 for all 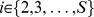, 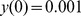 g, 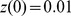, 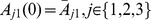, where 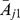 are the equilibrium concentrations in a tissue with 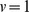, and all 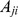 for 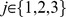 and 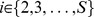 are left undefined until they arise via mutation. Mutations occur as discrete events, with one new mutant clone introduced at each event. Biologically, all mutation events except the first are assumed to be independent of time, the composition of the tumor and the number of previous mutation events. Mathematically we therefore assume that, if 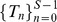 is the set of arrival times for the 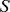 mutations defining new clones (assuming that 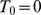), then 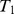, 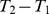, 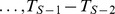 are independent, identically distributed exponential random variables with parameter 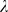 (mean 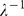; biologically, the mean interarrival times of mutations).

On the time intervals 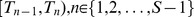, tumors grow according to models (1) and (11) with 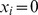 and 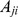 ignored for all 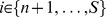 and 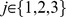. Mutant clones enter the model when they have grown to a size large enough to be buffered from stochastic extinction; therefore, a mutation event represents the arrival of an already sizable mutant clone, which is assumed to have initial mass of 0.1 mg and initial adenylate concentrations equal to the quasi-equilibrium for that clone at the current vascular density. So, at arrival times 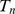, 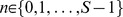, the mass of the new mutant clone, 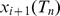, is set instantly to 0.1 mg, and the three adenlylate species for the new clone instantly take on their quasi-equilibrium values for that clone given 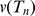; that is, 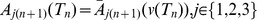. In the terminal time period, 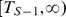, the tumor grows according to models (1) and (11), with all variables strictly positive. In practice, simulations ended at 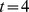 “years.” This horizon ensures that tumors, if viable, will grow beyond the model's design, given that the model is meant to represent tumors still growing in their exponential phase. In addition, it allows vascular density to equilibrate so that the evolutionarily dominant clone can more easily be defined (see below).

In any given simulation, only one parameter, either 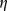 or 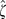, was allowed to evolve—all others were fixed at their default values. Values of 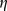 for each clone were drawn from the interval 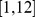 and for 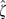 from the interval 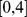, which includes essentially all biologically feasible values for these parameters [17]. The probability distributions for the draws were uniform over those intervals because, as mentioned earlier, single mutations may have large phenotypic effects, and a comprehensive mapping between pathological genetic and epigenetic alterations and phenotypic effects is unavailable. Given this uncertainty, a uniform probability distribution is the proper prior assumption. Further uncertainty arises in the mutation rate parameter, 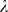. We explore the consequences of this uncertainty by fixing 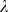 in each simulation, but varying it between 10 and 50 hours among simulations. In each case, however, the number of (eventually) competing clones, 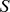, was fixed at 50. Each simulation scenario was then repeated 1000 times with the same initial conditions.

We evaluated the evolutionary outcome of the simulation using two measures. The first represents what would appear be the evolutionary “winner” in a histopathology study. In this case, we defined the “evolutionarily dominant,” or just dominant, clone to be the clone with the largest mass at the end of the simulation (4 years). The second measure of evolutionary success conforms more closely to an evolutionary biologist's notion of evolutionary advantage. In this case, we define the dominant clone as that which has the highest per capita growth rate at simulation's end. These two methods frequently identified different evolutionarily favored clones. For instance, many simulated tumors had small, but very rapidly growing, clones within them at the end of the simulation. These clones were clearly outcompeting all others, but because they arose late the the natural history of the tumor, they had not had time to impact the tumor's histology. This phenomenon lead us to fix 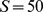 mutants—mutants that arise beyond the 50th almost always remain pathologically irrelevant.

A measure of clonal diversity within a tumor provides a concise description of a tumor's evolutionary state with, potentially, clinically relevant predictive power [1], [35], [36]. We therefore measure phenotypic diversity with the Shannon Diversity Index, or Shannon Information measure [37], which quantifies the mean relative abundance of tumor clones. Maley et al. [35] used it to measure clonal diversity in Barrett's esophagus, and we adopt it here for the same reason they did—it does not overemphasize the most common clone. Given that selection acts on clones of all sizes, and that rate of evolution depends on availability of adaptive phenotypes [38], Shannon's diversity index is a better measure of evolutionary potential than are many other commonly used indexes, like Simpson's. In the model, the practical difficulties of estimation from field data [39] are eliminated. The index, 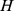, is defined as
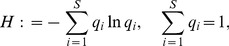
(12)where 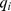 is the proportion of the tumor mass contributed by the 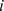
th clone. The Shannon index thus varies between 0 (a monomorphic tumor) and 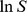 (all 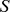 clones contribute equally to tumor mass). If the evolutionary endpoint is a monomorphic tumor, the Shannon index may be used as a rough measure of how close the tumor is to its climax histology (in the sense of the ecologist's “climax ecosystem”).

## Results and Discussion

### Evolution of Proliferation

**Figure 2:**
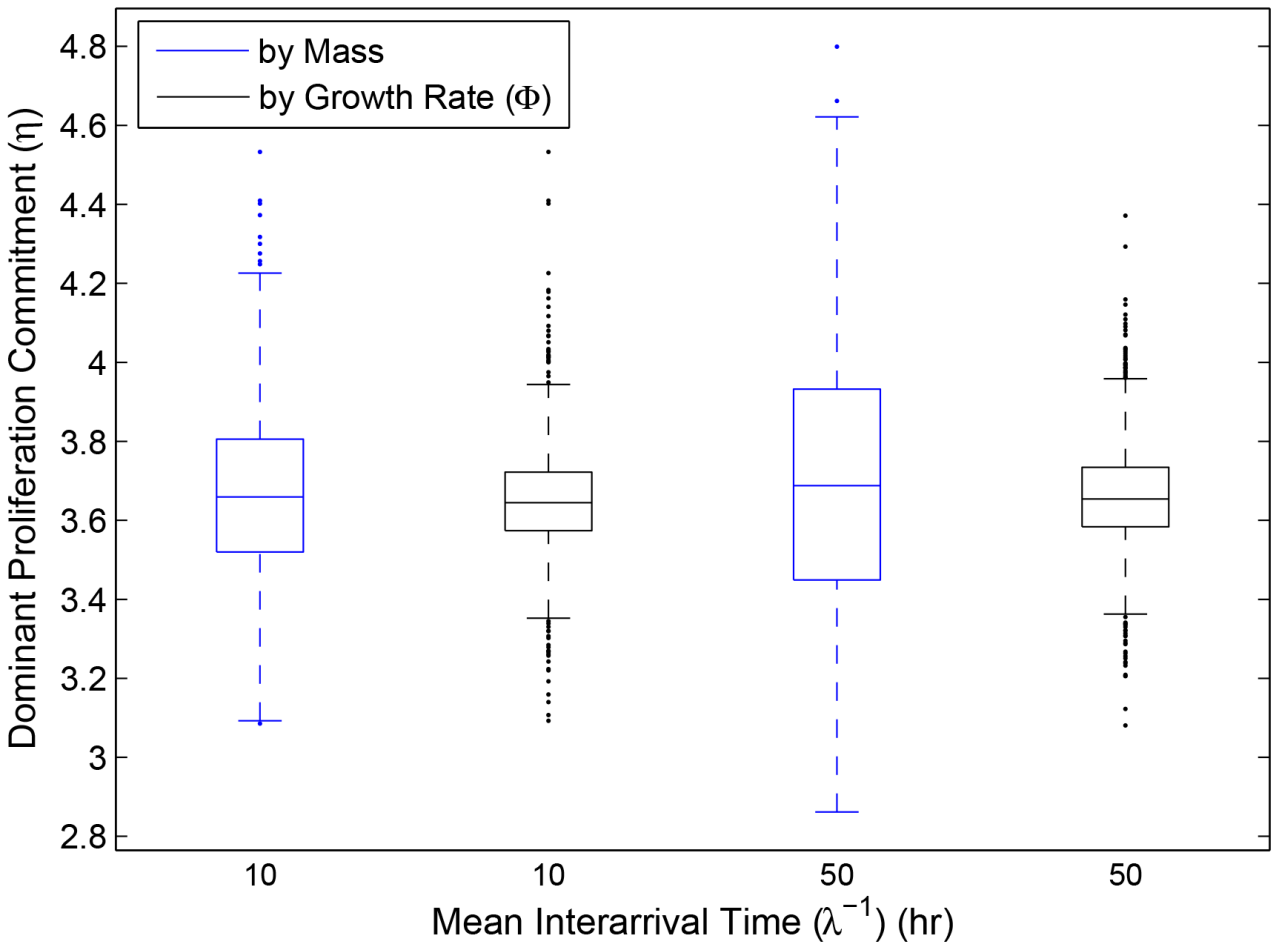
Evolutionarily “dominant” proliferation commitment (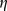) in 1000 simulations for each of two mutation rates: 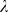 = 0.1 and 0.02 (plotted as mean interarrival time between mutations 10 and 50 hours, respectively). All parameters except 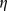 were set to the defaults in [17]. Shown are distributions of the histopathologist's “dominant” clone (clone with the most mass; blue) and the evolutionary biologist's dominant clone (clone with the largest per capita growth rate; black).

**Figure 3:**
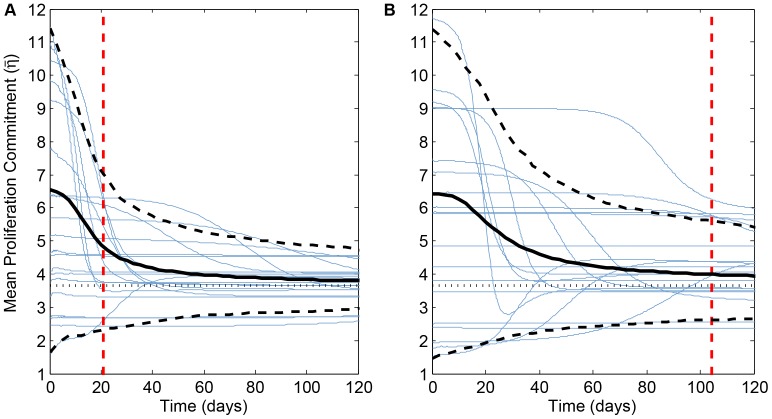
Change over time in weighted mean proliferation effort, 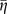 (see equation (13)). Blue curves in both panels represent the first 20 of the 1000 simulations plotted in Fig. 2. Solid black curves are the ensemble averages of all 1000 runs, dashed black curves mark the inner 95th percentile range for all runs, and dotted black lines represent the ESS predicted by adaptive dynamics theory (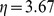 min^−1^). Dashed red lines represent mean time of the final mutation (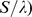. (A) 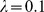; (B) 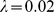.

Our simulations show that relaxation of restrictive adaptive dynamics assumptions do not alter the predictions of the adaptive dynamics analysis. No matter which definition one uses for “evolutionary dominance,” either the histopathologist's (most mass) or the evolutionary biologist's (largest per-capita growth rate), the mean dominant 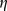 from our simulations agrees very well with the CSS (evolutionary attracting strategy) predicted by adaptive dynamics; for example, with default parameter values, the predicted ESS for proliferation effort was 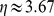 min^−1^ (approximately 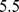 fmol or 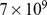 ATP molecules per minute in a resting cell, about half the rate measured in mouse LS cell culture [40]) which agrees very well with our simulations (Fig. 2). Nevertheless, the evolutionarily dominant clone at the close of any given simulation varies, sometimes significantly, from the theoretical ESS from adaptive dynamics. These deviations appear to be caused by mutational contingency—although the ESS is a deterministic evolutionary endpoint, by chance, the mutational history [26] fails to direct the system towards the optimal 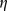 before the tumor grows out of bounds. This picture predicts that tumors of the same type, even in the same genetic background, will vary in evolutionary strategy in a symmetric distribution around the ESS (Fig. 2). However, at the whole-tumor level, the weighted mean proliferation effort, defined as
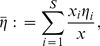
(13)also tends towards the predicted ESS, as does the ensemble average of many simulations (Fig. 3).

Historical contingency has a greater impact on the histopathological picture of tumor natural history than it does on the evolutionary view. If mutant clones nearest the ESS arise late, they will tend to contribute little to the tumor mass as it approaches lethality; the clone favored by selection has no time to become histologically significant. However, these favored clones' per capita expansion rates are large. Therefore, the variance in “dominant phenotype” will tend to be greater in the histopathological rather than the evolutionary view (Fig. 2). This effect is magnified as mutation rate decreases—longer mutation interarrival times gives early-arising, suboptimal clones more time to gain bulk before more well-adapted mutants crop up. As a result, if the number of mutations is fixed, tumors with lower mutation rates have greater variance in “dominant phenotype” at the histological level (Fig. 2, blue plots) and take longer to approach the ESS at the whole tumor level (Fig. 3). In contrast, since time of arrival has little effect on per capita growth rate, mutation rate has little to no effect on the “dominant” clone as defined by per capita growth rate (Fig. 2, black plots).

**Figure 4:**
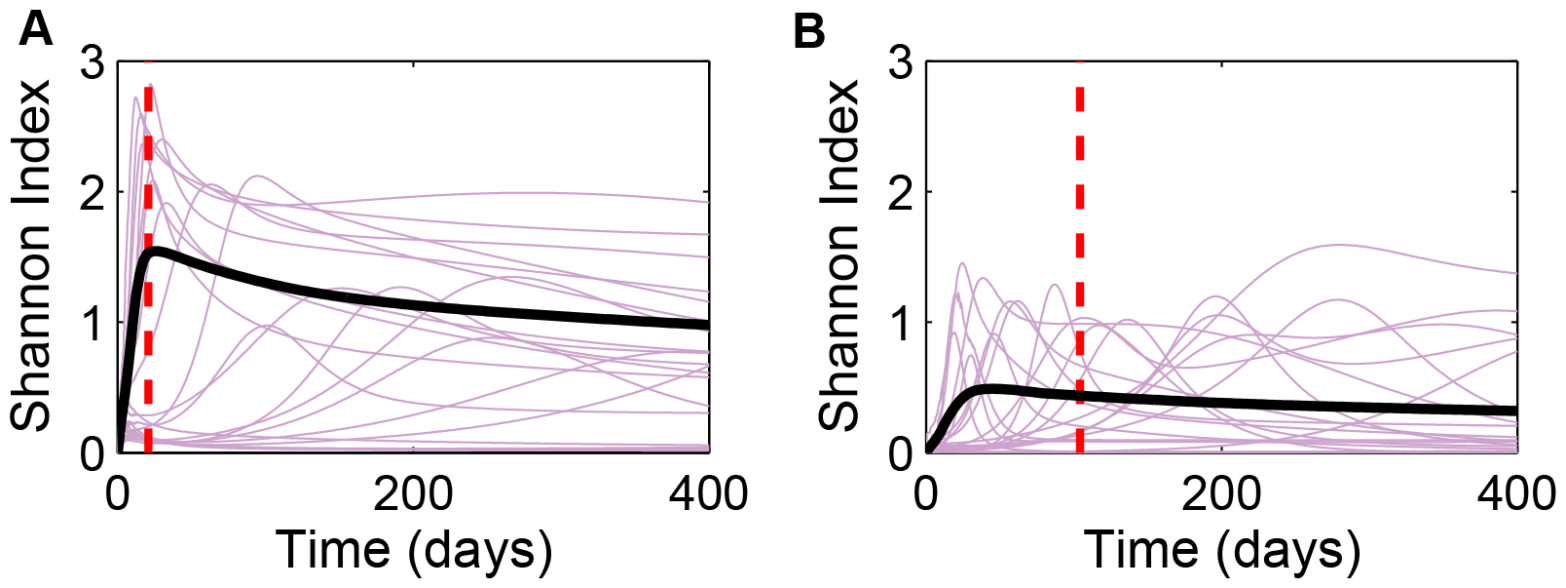
Dynamics of the Shannon diversity index, 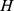, of individual tumors evolving in proliferation commitment, 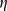. Purple curves in both panels represent the first 20 of the 1000 simulations plotted in Fig. 2. Solid black curves are the ensemble averages of all 1000 runs. Dashed red lines represent mean time of the final mutation (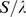). (A) 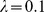; (B) 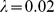.

These results suggest that, in clinical applications, simple measures of clonal diversity that fail to take clonal abundance into account, like mean phenotype or the ecologist's “species diversity” measure [39], will be inferior to metrics like the Shannon diversity index, which magnifies the relative contribution of rare clones to overall tumor diversity. We explore the consequences of this suggestion by evaluating the dynamics of diversity in our simulations, taking proliferation commitment, 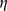, as the phenotype (Fig. 4). In most simulations, transient dynamics in 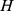 are longer than are transients in mean 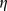 (compare Figs. 3 and 4). Interestingly, the Shannon index tends to increase with increasing mutation rate even though the total number of mutations remains the same (Fig. 4). Historical contingency is the culprit here, too. When interarrival times are relatively long, new mutant clones have a diminishing impact on this diversity measure since previously successful clones have more time to gain mass before they are challenged by new mutants. In addition, 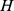 exhibits a large variance both among tumors in the ensemble and within a given tumor over time well after mutations have stopped (Fig. 4, purple curves).

How this variation relates to clinical prognosis is an interesting open question. Maley et al. [35] addressed a similar issue in Barrett's esophagus. Their study suggested that higher Shannon diversity index in cell ploidy predicts progression from the premalignant state to adenocarcinoma. These authors suggest that this correlation is causative since higher genetic diversity generally leads to more rapid evolution. In the context of the current study, the evolutionary endpoint is the ESS. Therefore, the same reasoning suggests that higher diversity should lead to more rapid convergence to that endpoint. Since diversity increases with mutation rate even for a fixed number of mutations, we therefore expect more rapidly mutating tumors to more rapidly approach the ESS. Indeed, the simulations predict precisely this pattern (Fig. 3). The significance of this is two-fold. Clonal diversity not only helps predict probability of cancer progression, it can also be used to assess the evolutionary potential of tumors that are already malignant. Therefore, we suggest that phenotypic diversity can be a clinically relevant measure that can complement genomic and genetic profiles which are at times so complicated by underlying genetic instability that they can obscure our understanding of tumor drivers.

### Evolution of the Angiogenic switch

As described in the introduction, the Nagy-Armbruster model [17] predicts that any ESS for angiogenesis effort (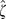) is an evolutionary repeller—rare mutant strategies further from the ESS are favored. Selection therefore pushes mean phenotype away from the ESS, resulting in runaway selection for either vascular hyper- or hypoplasia. This phenomenon is caused by a complex interaction between adenylate metabolism, energy charge homeostasis and vascular response to angiogenesis signaling. The angiogenesis ESS is also highly sensitive to proliferative effort. For example, if 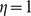, representing proliferative effort in a healthy, homeostatic tissue, the ESS 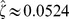 min^−1^ (about 0.08 fmol or 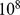 ATP molecules per min per cell). However, as 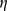 changes towards its ESS of 3.67 (assuming it does so independently of selection on angiogenesis potential), the ESS for 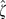 jumps past 90 (more than 135 fmol or 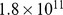 ATP molecules per minute per cell), beyond what is physiologically reasonable.

**Figure 5:**
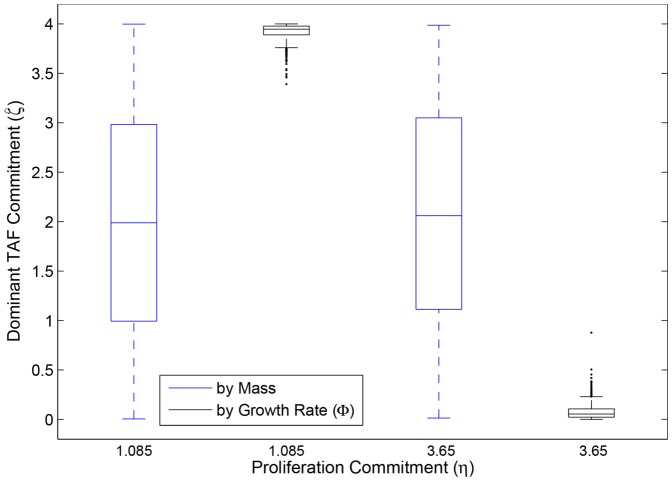
Evolutionarily dominant tumor angiogenesis factor commitment (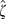) in 1000 simulations for two values of proliferative effort (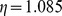 min^−1^ and 3.65 min^−1^). In both cases, 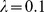. All other parameters except 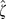 were set to defaults from [17]. (Compare Fig. 2.)

Here, as in the previous section, we relax the adaptive dynamics assumptions of one mutant challenger at a time and small mutational effect, although we retain the assumption that selection acts only on angiogenesis effort. Here again, simulations agree with the adaptive dynamics analysis. There exists an evolutionarily repelling ESS, so angiogenic commitment evolves to one extreme or the other, as can be seen in Fig. 5. If proliferation commitment is low, the angiogenesis ESS is also relatively low. In consequence, many mutants have an angiogenic commitment above the ESS. Because the ESS is repelling, those clones with the highest angiogenic commitment have the greatest advantage, and so 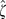 evolves to its highest possible value (Fig. 5, left-hand black box). However, if proliferation commitment is high, then the angiogenesis ESS is beyond what is physiologically possible but is still repelling. Therefore, mutants with the lowest angiogenic commitment are most favored, and 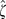 evolves to extremely low values (Fig. 5, right-hand black box).

**Figure 6:**
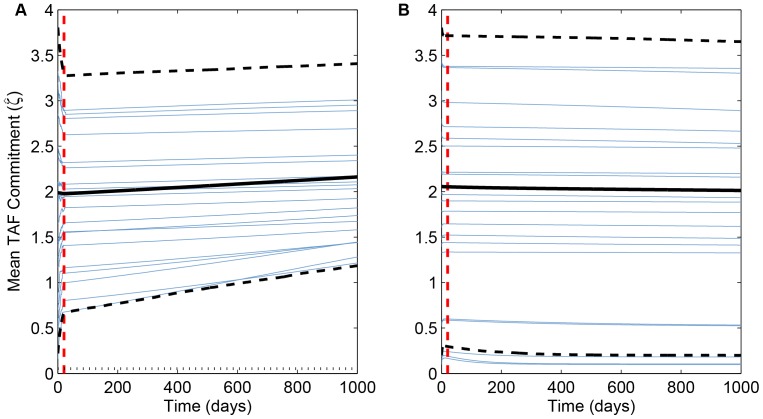
Change over time in weighted mean angiogenesis effort, 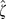. Blue curves in both panels represent the first 20 of 1000 simulations plotted in Fig. 5. Solid black curves are ensemble averages of all 1000 runs, dashed black curves are the inner 95th percentile of all runs, and dashed red lines are mean time of the last mutation. (Compare Fig. 3.) (A) 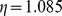 min^−1^; dotted horizontal line is the ESS from adaptive dynamics (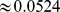 min^−1^). (B) 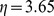 min^−1^; the ESS value of 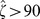 min^−1^ is not shown.

Nevertheless, these evolutionary forces remain clinically insignificant because historical contingency completely dominates the dynamics. By the end of the simulations, when tumors are well beyond lethal size, strategies that dominate by mass vary greatly in angiogenic phenotype, 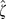 (Fig. 5, blue boxes). Therefore, even detailed histopathology studies would reveal no evolutionary pattern unless specific markers for angiogenesis were correlated with proliferation rate. This situation arises because the selection gradient for angiogenesis effort is extremely shallow [17]. Although extreme values of 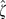 are favored, selective benefits of these extremes are tiny compared to disfavored strategies. Therefore, tumors tend to be dominated histologically by the initial clones that arise because the time required by any selectively favorable clone to overcome these early clones extends well beyond the time required for the tumor to grow to lethal size. Nevertheless, the general evolutionary trend towards “hypertumors” predicted by adaptive dynamics is still evident. All simulations show a clear trajectory towards either vascular hyper- or hypoplasia. In particular, tumors with low proliferation commitment (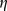 = 1.085 min^−1^) always evolve towards higher angiogenic potential (Fig. 6A), while tumors with high proliferation commitment (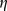 = 3.65 min^−1^) always creep towards failing angiogenesis (Fig. 6B).

**Figure 7:**
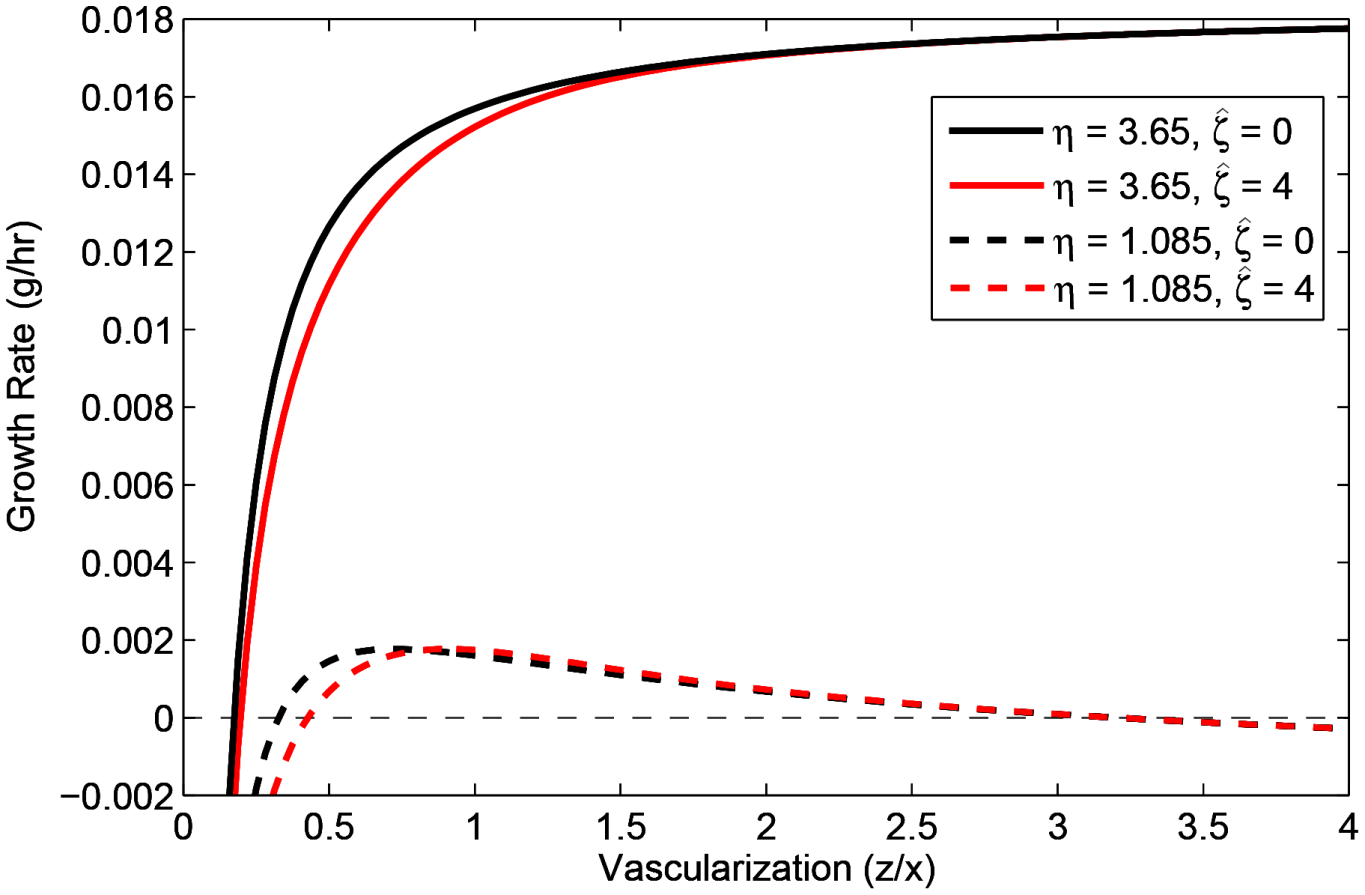
Per capita growth rate of various strategies against tumor vascularization. Gray, dashed horizontal line represents zero growth rate. Solid lines represent clones with high proliferation commitment (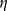 = 3.65 min^−1^), while dashed lines represent clones with low proliferation commitment (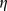 = 1.085 min^−1^). Black lines represent the lowest angiogenic clones (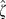 = 0 min^−1^), red lines; the highest angiogenic clones (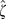 = 4 min^−1^), which encompasses the possible curves of all intermediate angiogenic clones.

In the former case, tumor necrosis occurs once tumor vascular density exceeds 3.2 times normal tissue vascularization (Fig. 7, dashed curves). The necrosis arises as selection continues to favor the most angiogenic clones in the (hypervascular) tumor; the energy wasted by massive angiogenic factor secretion in an environment that promises no more proliferative advantages to cells from more microvessel density ends up causing the tumor's downfall. In contrast, the latter case represents a “classical” hypertumor [14] as selection favors the least angiogenic clones, eventually forcing the vascular density below that required to sustain the cells. Specifically, tumors with proliferation commitment anywhere between 0 and 4 min^−1^ become necrotic once tumor vascularization drops below 0.2 (20% of normal vascular density; Fig. 7, solid curves).

**Figure 8:**
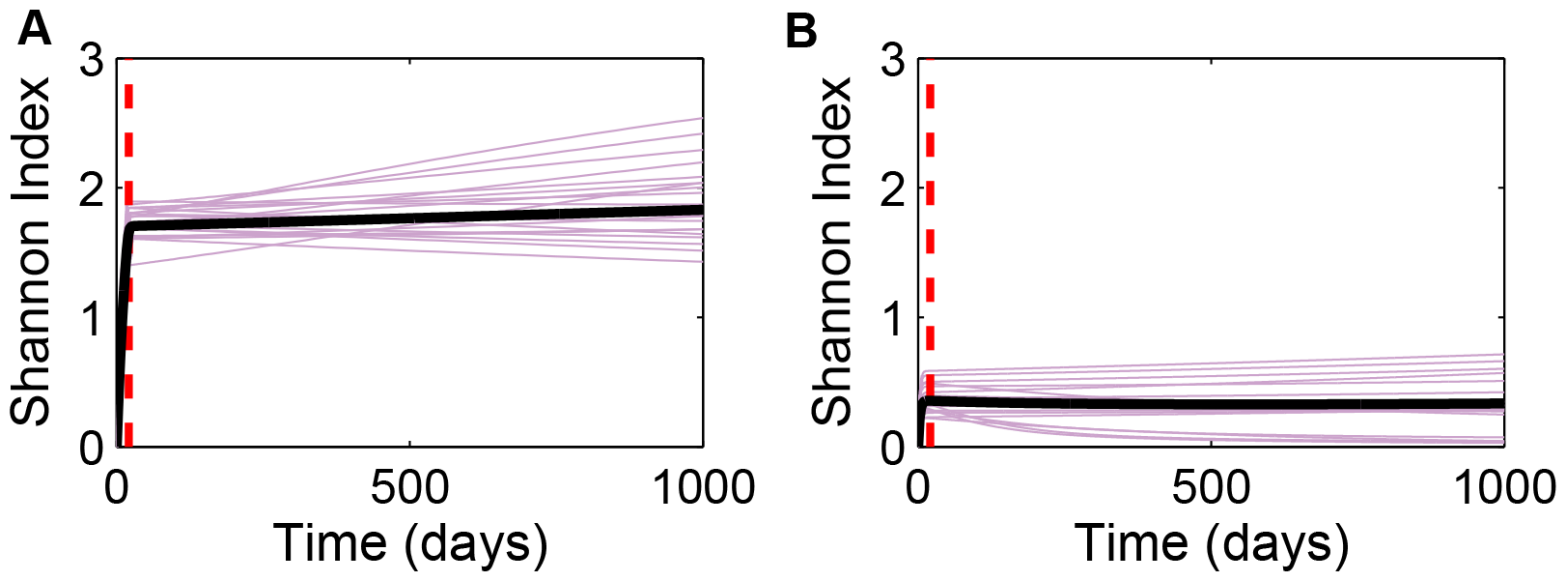
Dynamics of the Shannon diversity index, 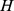, of individual tumors evolving in angiogenic commitment (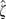) for two different constant proliferation commitments: (A) 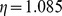 min^−1^; (B) 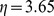 min^−1^. Purple curves in both panels represent the first 20 of the 1000 simulations plotted in Fig. 5. Solid black curves are the ensemble averages of all 1000 runs. Dashed red lines represent mean time of the final mutation (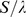), with 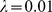.

As tumors progress towards their evolutionary endpoint—the ESS in the case of proliferative potential and extreme hypo- or hyperplasia for angiogenic ability—clonal diversity will tend to decrease. Therefore, the diversity index provides at least a rough measure of how close the tumor system is to its evolutionary endpoint. However, the picture is muddied because selection pressures on proliferation and on angiogenic capacities interact. Weak selective pressures on proliferation allow for increasing diversity in angiogenic capacity, whereas tumors evolving rapidly to a high proliferation commitment tend to be significantly less diverse (Fig. 8). Indeed, a small subset of highly proliferative tumors exhibit rapid declines in tumor diversity (Fig. 8B); no similar behavior was observed in tumors with low proliferation commitment (Fig. 8A).

Given the dominant role historical contingency plays in evolution of angiogenesis capacity in these simulations, the clinical significance of hypertumors remains an open question. Evolutionary suicide may be the ultimate endpoint of angiogenic tumors, but they may also tend to kill the host before that endpoint is approached in many, perhaps most, cases. However, the simulations as formulated here cannot be used to assess this suggestion since the parameterization is focused on early tumor growing in their exponential phase.

## Conclusions

Tumors exist not as homogeneous entities with universal properties or traits, but rather as diverse collections of heterogeneous cell lineages competing for resources with one another and surrounding healthy cells within the tumor stroma. Thus, viewing tumor progression as an evolutionary process is vital to understanding and eventually treating tumors so that resistance does not evolve. Previous adaptive dynamics modeling has shown that selection acts on cells' commitments to proliferation and TAF secretion potential based on their costs and benefits, defined primarily by their effects on metabolism and per capita growth rate [17]. However, the analytical techniques used in that study, based on adaptive dynamics, assumes certain biological constraints not commonly observed in cancer growing *in vivo*, including low phenotypic diversity and small mutational effects. Using numerical simulations, we relax these assumptions and show that diverse, complex tumors still adhere to the evolutionary pathways predicted by adaptive dynamics. In particular, (i) the ultimate evolutionary attractor for proliferative commitment is a finite ESS that is also convergence-stable, (ii) selection on angiogenic potential generates an ESS that becomes an evolutionary repeller, and therefore (iii) selection on angiogenesis potential produces vascular instability ultimately leading to evolutionary suicide (hypertumor) by inducing either vascular hypo- or hyperplasia. However, evolutionary trajectories in these simulations are so highly influenced by the tumor's specific mutational history that the predicted evolutionary endpoints may be largely irrelevant to tumor natural history *in vivo*.
